# 

**Published:** 1898-11

**Authors:** 


### CONTENTS:

### SELECTIONS.

Article I.—Cataphoresis. Arthur Holbrook, D. D. S ........... 289

" II.—The Importance of Establishing a Technic as well as Literary Standard for College Entrance. S. H. Guilford, D. D. S., Ph. D ........... 302

" III.—Causes of Dental Caries and Prophylaxis. J. Milton Rosenthal, D. D S ........... 308

" IV.—Pyorrhea Alveolaris—With some Notes From the Practice of the Author. A. W. Harlan, A. M., M. D. D. D. S ........... 314

" V.—Disease of the Peridental Membrane. C. M. Palmer, D. D. S ........... 320

" VI.—Pointing Instruments. W. H. Steele ........... 326

" VII.—Professional Recruits. Dr. Jonathan Taft ........... 328

### MONTHLY SUMMARY.

Taking Impressions ........... 330

Carious Dentin ........... 330

Gold-Shell Crown ........... 330

Ichthyol ........... 331

The Best Styptic ........... 331

Using Clamps ........... 332

Soldering Teeth ........... 332

Destroying Pulps Without Arsenic ........... 333

Counting Blood Corpuscles ........... 333

Trimming Vulcanite ........... 333

Operative and Prosthetic Pointers ........... 334

To Give Fine Finish to Gold ........... 334

Soap on Dam and Disk ........... 334

Gutta-Percha as a Retainer of Crowns and Bridges ........... 335

Celluloid Matrices ........... 335

Not Hart ........... 335

To Deposit Paste in Cavity ........... 336

For Polishing Plate Work ........... 336

Phorrhea ........... 336

